# Associations between Psychosocial Variables, Availability of Physical Activity Resources in Neighborhood Environment, and Out-of-School Physical Activity among Chinese Adolescents

**DOI:** 10.3390/ijerph18126643

**Published:** 2021-06-21

**Authors:** Nan Qiu, Xiaoli Gao, Xinge Zhang, Jialin Fu, Yechuang Wang, Rui Li

**Affiliations:** 1 Department of Healthcare Management, School of Health Sciences, Wuhan University, Wuhan 430071, China; 2013302170051@whu.edu.cn (N.Q.); fjl0708@whu.edu.cn (J.F.); ywang20@whu.edu.cn (Y.W.); 2 Faculty of Dentistry, National University of Singapore, Singapore 129800, Singapore; dengx@nus.edu.sg; 3 Saw Swee Hock School of Public Health, National University of Singapore, Singapore129800, Singapore; 4 Department of Medicine and Therapeutics, The Chinese University of Hong Kong, Shatin, New Territories, Hong Kong 999077, China; xinge@link.cuhk.edu.hk

**Keywords:** physical activity, adolescent, peer support, parental support, neighborhood environment

## Abstract

This study aimed to evaluate the relationship between psychosocial variables (peer support, parental support, autonomous motivation, and controlled motivation), availability of physical activity resources in a neighborhood environment, and out-of-school moderate to vigorous physical activity (MVPA) among Chinese adolescents. The questionnaire of Family Life, Activity, Sun, Health, and Eating (FLASHE) Study was used to collect information on demographics, socioeconomic status, psychosocial variables, available physical activity resources in the neighborhood environment, and minutes of out-of-school MVPA. ANOVA analysis and multiple regression analysis were performed. The mean age of the 3833 adolescents included in our analysis was 14.7 years old (SD = 1.7). Peer support (b = 9.35, 95% CI: 7.55–11.15), autonomous motivation (b = 6.46, 95% CI: 4.09–8.82), parental support (b = 3.90, 95% CI: 1.75–6.07), and availability of physical activity resources in neighborhood environment (b = 3.18, 95% CI: 1.99–4.36) were significantly associated with out-of-school MVPA (*p* < 0.05). Controlled motivation was insignificantly related to minutes of out-of-school MVPA. Boys spent more time on out-of-school MVPA than girls (*p* < 0.001) and had a high level of peer support, parental support, and motivation (*p* < 0.05). Our findings suggest that interventions targeting the out-of-school MVPA among Chinese adolescents should focus on the psychosocial variables and neighborhood environment.

## 1. Introduction

Physical activity (PA) is defined as any bodily movement produced by skeletal muscles that requires energy expenditure [1]. It refers to all movement including during leisure time, for transport to reach destinations, or as part of a person’s work [1]. Regular and adequate levels of PA play an essential role in improving health and quality of life. According to the World Health Organization (WHO), an adult should engage in at least 150 min of moderate-intensity PA throughout the week, or at least 75 min of vigorous-intensity PA throughout the week, or an equivalent combination of moderate- and vigorous-intensity activity [2]. In reverse, lack of PA can increase the risk of several chronic diseases, including obesity, cardiovascular disease [3], anxiety, and depression [4]. It has been reported that decreased moderate to vigorous physical activity (MVPA) was associated with an increased risk of all-cause mortality [5]. However, 28% of adults worldwide did not achieve the levels recommended by WHO [6].

Recently, the lack of PA in adolescence has become a global concern [7]. Puberty was identified as the age of greatest decline in PA [8]. WHO recommends that children and adolescents aged 5–17 should accumulate at least 60 min of MVPA every day [2]. However, a study conducted in 2016 found that 81% of adolescents aged 11–17 years worldwide were physically inactive [9]. Another study involving 72,845 schoolchildren from 34 countries reported that only 23.8% of boys and 15.4% of girls met the recommendation [10]. In China, less than one-third of adolescents accumulate 60 min of MVPA every day [11,12]. 

Teenagers were more active in out-of-school hours than school hours [13,14]. A study on Chinese secondary school students found that about 70% of youth participated in out-of-school MVPA [15]. Out-of-school time was thus considered as a period that could offer potential opportunities to increase MVPA levels for adolescents [16,17]. It has been suggested that adolescents who participate in MVPA outside of school have better body composition, flexibility, and cardiorespiratory endurance [18]. However, current studies mostly focus on overall PA and PA at school; factors that influence out-of-school MVPA levels in adolescents need to be determined so that effective policies and interventions can be implemented. 

Social networks could affect personal health-promoting behaviors [19]. Social support generally refers to any behavior from other people such as parents or friends that assists an individual in achieving desired goals [20]. Social support can influence PA through instrumental and direct (transportation), emotional and motivational effects (encouragement, praise), or observational effects (modeling) [21]. It has been shown that peer support and parental support were significantly related to MVPA level among 8th- and 11th-grade students in the US [22]. However, Gillison et al. reported that there was no evidence of a moderation effect of parental support on PA time in boys [23]. Self-determination theory (SDT) maintains that motivation, including autonomous motivation and controlled motivation, are predictors of human behaviors. Autonomous motivation refers to people identify with an activity’s value and ideally will integrate it into their sense of self. In contrast, controlled motivation means a person’s behavior is affected by external and internal rewards or punishments [24]. A previous study has found that autonomous motivation is a critical factor in promoting PA [25]. Therefore, these psychosocial variables may play an essential role in modulating the MVPA level. On the other hand, the availability of PA resources in a neighborhood environment (e.g., availability of recreation facilities) is also likely to influence PA among adolescents [26]. However, a systematic review including 169 studies reported that the relationship between neighborhood environment and PA among children and adolescents was uncertain compared with adults [27]. 

Thus, this study aims to investigate the relationship between psychosocial variables (peer support, parental support, autonomous motivation, and controlled motivation), availability of PA resources in a neighborhood environment, and minutes of out-of-school MVPA among Chinese adolescents.

## 2. Methods

### 2.1. Participants

A cross-sectional survey was carried out at a school in Wuhan, China, in October 2019. Before participation, informed consent forms were sought from all adolescents (*n* = 4027) aged 10–20 years, and their parents provided assent. The study was conducted in accordance with the Declaration of Helsinki, and all procedures were reviewed and approved by the Wuhan University Ethics Board (ethical approval code: 2019YF2056) and the local school district administrators. A total of 4027 adolescents were fully eligible and consented to participate in the study to provide information on sociodemographic variables, psychosocial characteristics, available PA resources in neighborhood environment, and out-of-school MVPA minutes. Participants filled in the questionnaires by themselves, and all of them returned the questionnaires. Questionnaires, which had missing data in out-of-school MVPA minutes, were excluded. Finally, 3833 participants were included in the analytical sample.

### 2.2. Measures

Adolescents reported their age, gender, grade, ethnicity, weight, height, living area, family monthly income, and parents’ educational background. The questionnaires of the Family Life, Activity, Sun, Health, and Eating (FLASHE) Study were used to collect information on the psychosocial variables and available PA resources in the neighborhood environment. The FLASHE Study was developed by the National Cancer Institute with cognitive testing and usability testing. A cross-sectional study based on this questionnaire was conducted in the United States between April 2014 and October 2014 [28].

Psychosocial variables included peer support, parental support, autonomous motivation, and controlled motivation. Items we used to assess each variable could be viewed in Appendix A Table A1. Participants selected how much they disagree or agree with each of the statements ranging from strongly disagree (score of 1), disagree (score of 2), neutral (score of 3), agree (score of 4), and strongly agree (score of 5). We calculated the average scores of each psychosocial variable for analysis. 

The availability of PA resources in neighborhood environment within a 10–15 min-walk in any direction was assessed by the following list: (1) indoor recreation or exercise facility; (2) school with recreation facilities open to the public; (3) bike/hiking/walking trails, paths; (4) basketball courts, running track/other playing fields (such as football, tennis, skate park, etc.); (5) public park. Response options were dichotomized as “yes/no”, and neighborhood environment score was calculated as the total number of “yes” (0−5). 

Minutes of out-of-school MVPA in the past week were the main outcomes in this study. Five items of the self-reported Youth Activity Profile (YAP) were used to evaluate the levels of out-of-school MVPA throughout a week: (1) How many days did you do some form of physical activity before school, including activity at home NOT walking or biking to school, for at least 10 min? (2) How many days did you do some form of physical activity after school for at least 10 min? (3) How many days did you do some form of physical activity in the school evenings for at least 10 min? (4) How much physical activity did you do last Saturday? (5) How much physical activity did you do last Sunday? The YAP questionnaire is developed for adolescents from 4th to 12th grade to provide feedback on students’ MVPA behavior [29]. The YAP items are scored on a 1–5 scale. Scores of weekdays and weekends are averaged, respectively, to reflect the composite raw score for MVPA. Separate calibration models are applied to each YAP section raw score. Predicted percent time in MVPA from the models can be converted to weekly minutes of activity by multiplying predicted percent MVPA by the respective section time in minutes. The YAP questionnaire has been validated against accelerometers in American teenagers [30]. 

The questionnaires had good reliability and validity: Cronbach’s alpha = 0.84, Kaiser–Meyer–Olkin = 0.88, *p*_Bartlett_ < 0.001.

### 2.3. Statistical Analysis

All statistical analyses were performed using SPSS Statistics 21.0 (IBM, Armonk, NY, USA). A multiple imputation approach was employed to maximize data availability when missing data met the assumption for missing at random. Descriptive statistics were carried out to describe the distribution of demographic, psychosocial, and neighborhood variables. Continuous variables were represented by mean and standard deviation (SD) (normal) or median and interquartile range (non-normal), while subtype variables were represented by frequency and percentage. Differences between genders (male/female) and education stages (secondary school/high school) in mean levels of out-of-school MVPA minutes, peer support, parental support, autonomous motivation, controlled motivation, and neighborhood environment were examined using ANOVA analysis or Wallis test. A series of multiple linear regression models using the STEPWISE method were run to test the associations between psychosocial variables (peer support, parental support, autonomous motivation, and controlled motivation), neighborhood environment, and out-of-school MVPA. Possible confounders including gender, grade, body mass index z-score, ethnicity, living area, family monthly income, and parents’ educational background were controlled. Meanwhile, we performed collinearity diagnostics, including examination of the correlation coefficient matrix and variance inflation factors (<5), to determine a final model. Unstandardized coefficients and related 95% confidence intervals were reported. All *p*-values were two-tailed, and *p*-values < 0.05 were considered statistically significant.

## 3. Results

The descriptive statistics of participants are presented in Table 1. A total of 3833 students with an average age of 14.7 years old (SD = 1.7) were included in our analysis. Of those, 2016 (52.6%) were males and 1776 (43.4%) were secondary school students. Most participants were Han and lived in an urban area. A minority of participants’ family monthly income was less than 5000 RMB. About one-third of adolescents did not have any out-of-school MVPA on weekdays. 

Table 2 presented the mean time spent in out-of-school MVPA, and mean scores of peer support, parental support, autonomous motivation, controlled motivation, and availability of PA resources in neighborhood environment by gender and educational level. Boys spent more time on out-of-school MVPA than girls (312.17 min/week vs. 274.85 min/week, *p* < 0.05) and had higher scores in peer support, parental support, autonomous motivation, and controlled motivation (*p* < 0.05). Secondary school students participated in more minutes of out-of-school MVPA (336.71 min/week vs. 252.68 min/week, *p* < 0.05) and scored higher in peer support and parental support, and lower in the availability of PA resources in neighborhood environment (*p* < 0.05), as compared with high school students.

Table 3 presented the results of multiple regression analysis for out-of-school MVPA. After adjusting for sociodemographic variables (gender, grade, ethnicity, living area, paternal education level, and family monthly income), a series of multivariable linear regression models were performed to examine the associations between neighborhood environment, psychosocial variables, and out-of-school MVPA. Peer support (b = 9.35, 95% CI: 7.55–11.15), parental support (b = 3.90, 95% CI: 1.75–6.07), autonomous motivation (b = 6.46, 95% CI: 4.09–8.82), and availability of PA resources in neighborhood environment (b = 3.18, 95% CI: 1.99–4.36) were all significantly associated with times of out-of-school MVPA among Chinese adolescents (*p* < 0.05). Grade (b = −28.35, 95% CI: −29.50, −27.19) and female (b = −7.19, 95% CI: −11.3, −3.14) were negatively correlated with out-of-school MVPA among adolescents (*p* < 0.05). Controlled motivation was insignificantly related to minutes of MVPA outside of school. Interestingly, there was no significant association between the availability of PA resources in neighborhood environment and out-of-school MVPA among girls on weekdays, as shown in Appendix A Table A2. The respective results of boys and girls in secondary school and high school can be viewed in Appendix A Table A3.

## 4. Discussion

Encouraging adolescents to participate in out-of-school MVPA is an important strategy for health promotion. The findings of this study shed some light on the associations between psychosocial factors (peer support, parental support, autonomous motivation, and controlled motivation), availability of PA resources in neighborhood environments, and out-of-school MVPA among Chinese adolescents. 

There was a significant association between peer support and out-of-school MVPA. Adolescents who reported exercising with their peers spent more minutes on MVPA outside of school, which was also reported by other findings [31,32,33]. Exercising with peers could increase self-efficacy or enjoyment for engaging in MVPA among teenagers [34]. Moreover, according to the expectancy-value theory, encouragement from peers could support individuals’ feelings of competence or expectancies for participating in PA [35]. This suggests that encouraging adolescents to be active with their friends during the out-of-school period is helpful for the youth to achieve higher levels of MVPA. In addition, we found that boys received more PA-related support from their peers than girls, which was consistent with a previous study [33]. Notably, we observed a significant difference in peer support on out-of-school MVPA between secondary school students and high school students, which was contrary to a similar study [33]. This could be partly explained by the fact that high school students with more academic pressure are likely to increase the time on study actively or passively while reducing the out-of-school time spent on playing with their peers in China. 

A psychological study reported that when children moved into puberty, the influence of parents decreased, while the peers began to play a more important role [36]. However, it was shown in this study that parental support was still significantly related to the minutes of out-of-school MVPA, both in boys and girls. Previous studies using the same questionnaire also indicated that parental support was positively associated with MVPA in American youths aged 9–18 years old [37,38]. Furthermore, a longitudinal study identified that parental support reported by teenagers in early adolescence had long-term associations with MVPA up to 5 years later [39]. 

Autonomous motivation was positively associated with out-of-school MVPA minutes. A recent study using the YAP questionnaire in 12–17-year-old teenagers also reported that autonomous motivation variables could positively predict out-of-school MVPA on weekdays and weekends [40]. However, the relationship between controlled motivation and out-of-school MVPA was insignificant in the present study, given that the willingness to exercise depended on self-positive regulation but not on negative emotions or external cues [41,42]. Autonomous motivation, rather than controlled motivation, could mediate the effect of goals on PA in adolescents [43]. Therefore, we should pay more attention to enhancing autonomous motivation on engaging in MVPA in adolescents. Interventions on promoting MVPA will be effective if the youth are aware of the importance of exercise.

Adolescents who had more available PA resources, such as exercise facilities, paths, playing fields, and parks, in their neighborhood environment spent more time on MVPA outside of school [44]. However, PA resource availability in the neighborhood environment was not significantly related to out-of-school MVPA levels among girls during weekdays in the present study. We speculated that this phenomenon was affected by the low level of out-of-school MVPA among girls during weekdays. Minimally active adolescent girls prefer to exercise in their home environment rather than in the neighborhood environment [45]. 

Participants in this study reported an insufficient level of engagement in out-of-school MVPA, especially on weekdays. Particularly, boys spent more time on out-of-school MVPA than girls [31], and higher grade students engaged in less out-of-school MVPA than lower grade students [32]. Girls and high school students are potential target groups for intervention.

There are several limitations in the present study. Firstly, though we had a relatively large sample size, participants in this study were recruited from one school, which limited the representativeness of the study. Secondly, we used the self-reported MVPA minutes of adolescents, which may lead to bias. Lastly, the finding of this research could only support the associations but not causality, given its limitation as a cross-sectional study.

## 5. Conclusions

Psychosocial variables (peer support, parental support and autonomous motivation) and availability of PA resources in neighborhood environment are positively related to out-of-school MVPA. It can be effective to promote MVPA in Chinese adolescents by encouraging adolescents to exercise with their peers, enhancing their autonomous motivation to participate in exercise, receiving encouragement and help from parents, and creating a conducive neighborhood environment.

## Figures and Tables

**Table 1:** Characteristics of participants *.

	Male	Female	Total
	*n* (%) (*n* = 2016)	*n* (%) (*n* = 1777)	*n* (%) (*n* = 3833)
Education stages			
Secondary school	936 (46.4)	820 (46.2)	1776 (46.3)
High school	1080 (53.6)	957 (53.9)	2057 (53.7)
Ethnicity			
Han	1985 (98.5)	1752 (98.6)	3737 (97.5)
Ethnic minority	18 (0.9)	19 (1.1)	37 (1.0)
Living area			
Urban	1680 (83.4)	1470 (82.7)	3150 (82.2)
Rural	250 (12.4)	232 (13.1)	482 (12.6)
Family monthly income (RMB)			
<5000	253 (12.6)	243 (13.7)	496 (13.0)
5000–20,000	1383 (68.6)	1244 (70.0)	2627 (68.5)
>20,000	225 (11.2)	144 (8.1)	369 (9.7)
Father’s educational background			
Lower than bachelor’s	840 (41.7)	1327 (74.7)	2167 (56.6)
Bachelor’s or above	1120 (55.6)	405 (22.8)	1525 (39.8)
Mother’s educational background			
Lower than bachelor’s	1666 (82.6)	1447 (81.4)	3113 (81.2)
Bachelor’s or above	311 (15.5)	289 (16.3)	600 (15.7)
No out-of-school MVPA	99 (4.9)	263 (14.8)	362 (9.4)
No out-of-school MVPA on weekdays	407 (20.3)	765 (43.1)	1172 (30.6)
No out-of-school MVPA on weekends	128 (6.4)	364 (20.5)	492 (12.9)
Body mass index z-score, mean (SD)	0.20 (1.3)	0.31 (1.4)	0.08 (1.3)

Note: * Non-imputed data, hence the sample size varies by variable due to missing data. MVPA, moderate to vigorous physical activity; SD, standard deviation.

**Table 2:** Out-of-school MVPA, psychosocial variables, and neighborhood environment (mean and SD) by gender and educational levels.

	Total	Male	Female	*p*-Value	Secondary School	High School	*p*-Value
Out-of-school MVPA (min/week)	291.61 (73.68)	312.17 (71.26)	274.85 (70.71)	<0.001	336.71 (49.94)	252.68 (68.50)	<0.001
Peer support	3.51 (1.19)	3.75 (1.19)	3.24 (1.14)	<0.001	3.58 (1.17)	3.51 (1.19)	0.008
Parental support	3.13 (0.99)	3.17 (1.05)	3.09 (0.91)	0.012	3.23 (1.03)	3.13 (0.99)	<0.001
Autonomous motivation	3.47 (1.04)	3.56 (1.04)	3.28 (1.00)	<0.001	3.47 (1.05)	3.48 (1.04)	0.64
Controlled motivation	2.61 (0.96)	2.79 (0.99)	2.41 (0.87)	<0.001	2.59 (0.96)	2.60 (0.96)	0.57
Availability of PA resources in neighborhood environment	2.76 (1.68)	2.76 (1.73)	2.77 (1.61)	0.777	2.63 (1.64)	2.76 (1.68)	<0.001

Note: MVPA, moderate to vigorous physical activity; SD, standard deviation.

**Table 3:** Multiple regression analysis for out-of-school MVPA among adolescents.

	b (95% CI)	β	*p*-Value
Out-of-school MVPA			
Peer support	9.35 (7.55, 11.15)	0.15	<0.001
Parental support	3.90 (1.75, 6.07)	0.05	<0.001
Autonomous motivation	6.46 (4.09, 8.82)	0.09	<0.001
Availability of PA resources in neighborhood environment	3.18 (1.99, 4.36)	0.07	<0.001
Grade	−28.35 (−29.50, −27.19)	−0.62	<0.001
Gender ^1^	−7.19 (−11.3, −3.14)	−0.05	<0.001
Family monthly income	2.54 (0.45, 4.62)	0.03	0.017
Out-of-school MVPA on weekdays			
Peer support	4.57 (3.42, 5.72)	0.06	<0.001
Parental support	3.5 (2.0, 4.9)	0.03	<0.001
Autonomous motivation	4.73 (2.78, 5.96)	0.07	<0.001
Availability of PA resources in neighborhood environment	0.9 (0.2, 1.6)	0.01	0.016
Grade	−2.76 (−3.05, −2,47)	−0.07	<0.001
Gender	−5.21 (−7.22, −3.20)	−0.09	<0.001
Family monthly income	2.43 (1.40, 3.46)	0.07	<0.001
Out-of-school MVPA on weekends			
Peer support	12.2 (10.0, 14.4)	0.10	<0.001
Parental support	7.4 (4.9, 9.8)	0.04	<0.001
Autonomous motivation	11.6 (9.2, 13.9)	0.10	<0.001
Availability of PA resources in neighborhood environment	4.2 (2.8, 5.6)	0.02	<0.001
Grade	−17.1 (−21.7, −12.5)	−0.02	<0.001
Gender	−14.47 (−22.39, −6.56)	−0.06	<0.001

Note: MVPA, moderate to vigorous physical activity. b (95% CI), unstandardized coefficients and its 95% confidence interval. β, standardized coefficients. ^1^ Male = 1, Female = 2.

## Data Availability

The data presented in this study are available on request from the corresponding author. The data are not publicly available due to privacy restrictions.

## Appendix A

**Table A1:** Measures of the psychosocial variables.

**Psychosocial Variables**	**Items**
Peer support	1. My friends play sports or are physically active with me.
Parental support	1. My parent(s) have to make sure that I get enough physical activity. 2. My parent(s) take me places where I can be physically active. 3. My parent(s) and I decide together how much physical activity I have to do. 4. My parent(s) make me exercise or go out and play. 5. My parent(s) try to be physically active when I’m around. 6. It’s okay for my parent(s) to make rules about how much time I spend being physically active/playing.
Autonomous motivation	1. I have thought about it and decided that I want to exercise. 2. Exercise is an important thing for me to do.
Controlled motivation	1. Others would be upset with me if I did not exercise. 2. I would feel bad about myself if I did not exercise.

**Table A2:** Multiple regression analysis for out-of-school MVPA among girls.

	**b (95% CI)**	**β**	***p*-Value**
Out-of-school MVPA			
Peer support	6.89 (4.66, 9.11)	0.11	<0.001
Parental support	4.32 (1.34, 7.23)	0.05	0.004
Autonomous motivation	4.30 (1.67, 6.92)	0.06	0.001
Availability of PA resources in neighborhood environment	2.63 (1.12, 4.13)	0.06	0.001
Grade	−31.89 (−33.33, −30.45)	−0.74	<0.001
Father’s educational background	2.89 (0.26, 5.51)	0.03	0.031
Out-of-school MVPA on weekdays			
Peer support	4.66 (3.13, 6.19)	0.16	<0.001
Parental support	3.80 (1.83, 5.77)	0.10	<0.001
Autonomous motivation	5.17 (3.37, 6.97)	0.15	<0.001
Grade	−3.95 (−4.93, −2.96)	−0.20	<0.001
Out-of-school MVPA on weekends			
Peer support	6.62 (4.44, 8.81)	0.11	<0.001
Parental support	3.98 (1.17, 6.79)	0.05	0.006
Autonomous motivation	4.51 (1.48, 6.63)	0.06	0.002
Availability of PA resources in neighborhood environment	2.62 (1.14, 4.09)	0.06	0.001
Grade	−30.03 (−31.44, −28.61)	−0.73	<0.001
Father’s educational background	2.81 (0.24, 5.38)	0.04	0.032

Note: MVPA, moderate to vigorous physical activity. b (95% CI), unstandardized coefficients, and its 95% confidence interval. β, standardized coefficients.

**Table A3:** Multiple regression analysis for out-of-school MVPA among boys and girls in secondary school and high school.

	**b (95% CI)**	**β**	***p*-Value**
Boys in secondary school			
Peer support	4.31 (3.00, 5.62)	0.10	0.001
Autonomous motivation	9.27 (7.79, 10.75)	0.20	<0.001
Grade	−27.88 (−29.65, −26.10)	−0.45	<0.001
Boys in high school			
Peer support	13.21 (11.57, 14.86)	0.23	<0.001
Parental support	4.33 (2.42, 6.25)	0.06	0.024
Autonomous motivation	8.36 (6.08, 10.63)	0.12	<0.001
Controlled motivation	5.30 (3.07, 7.54)	0.07	0.018
Availability of PA resources in neighborhood environment	2.52 (1.46, 3.58)	0.06	0.017
Grade	−30.21 (−32.33, −28.09)	−0.37	<0.001
Family monthly income	7.54 (5.73, 9.35)	0.11	<0.001
Girls in secondary school			
Autonomous motivation	6.34 (4.90, 7.78)	0.14	<0.001
Father’s educational background	3.42 (1.86, 4.98)	0.07	0.028
Grade	−26.38 (−28.12, −24.61)	−0.48	<0.001
Girls in high school			
Peer support	8.76 (6.93, 10.59)	0.17	<0.001
Parental support	7.85 (5.43, 10.28)	0.16	0.001
Availability of PA resources in neighborhood environment	3.64 (2.37, 4.90)	0.10	0.004

Note: MVPA, moderate to vigorous physical activity. b (95% CI), unstandardized coefficients, and its 95% confidence interval. β, standardized coefficients.
